# Causes and Clinical Impact of Loss to Follow-Up in Patients with Proliferative Diabetic Retinopathy

**DOI:** 10.1155/2020/7691724

**Published:** 2020-02-08

**Authors:** Hazem Abdelmotaal, Walid Ibrahim, Mohamed Sharaf, Khaled Abdelazeem

**Affiliations:** Department of Ophthalmology, Faculty of Medicine, Assiut University, Assiut, Egypt

## Abstract

**Purpose:**

This study determined the clinical impact and causes of loss to follow-up (LTFU) from the patients' perspective in individuals with proliferative diabetic retinopathy (PDR) who received panretinal photocoagulation (PRP) and/or intravitreal injections (IVIs) of antivascular endothelial growth factor (VEGF).

**Methods:**

This prospective cohort study included 467 patients with PDR who received PRP and/or IVIs of anti-VEGF between May 2013 and June 2018. LTFU was defined as missing any follow-up visit for any interval exceeding 6 months, provided that patients eventually resumed care. Main outcome measures include rates and causes of LTFU.

**Results:**

A total of 391 patients (83.7%) were followed up, and 76 patients (16.3%) were LTFU over the study period. Rates of LTFU decreased with age (*P*=0.005). Questionnaire analysis conducted for patients' LTFU showed a significant positive correlation between best corrected visual activity (BCVA) loss and patient's lack of trust and satisfaction with treatment (rs = 0.458, *P*=0.005). Questionnaire analysis conducted for patients' LTFU showed a significant positive correlation between best corrected visual activity (BCVA) loss and patient's lack of trust and satisfaction with treatment (rs = 0.458, *P*=0.005). Questionnaire analysis conducted for patients' LTFU showed a significant positive correlation between best corrected visual activity (BCVA) loss and patient's lack of trust and satisfaction with treatment (rs = 0.458, *P*=0.005). Questionnaire analysis conducted for patients' LTFU showed a significant positive correlation between best corrected visual activity (BCVA) loss and patient's lack of trust and satisfaction with treatment (rs = 0.458,

**Conclusions:**

LTFU threatens vision in PDR patients receiving PRP and/or IVIs of anti-VEGF. Possibly, patient-specific LTFU causes should be addressed before treatment in order to minimize the risk of LTFU. The clinical trial is registered with NCT04018326 (trial registration: ClinicalTrials.gov Identifier: NCT04018326, 10th of July 2019 “Retrospectively registered”).

## 1. Introduction

Proliferative diabetic retinopathy (PDR) is a leading cause of vision loss in diabetic patients [1, 2], with approximately 1.5% of diabetic adults having PDR [3]. Panretinal photocoagulation (PRP) is currently the only successful, evidence-based treatment for PDR and results in a 50%–60% reduction in the risk of severe visual loss because of neovascularization regression during the first 3 months after treatment [4]. However, this treatment is associated with various adverse effects, such as increased risk of macular edema, peripheral field loss, and night vision loss [5]. Furthermore, even after successful PRP, a proportion of patients require additional laser treatment, and some of them also require pars plana vitrectomy (PPV) [6, 7]. The advent of intravitreal injections (IVIs) of antivascular endothelial growth factors (VEGF) has revolutionized the management of PDR. For example, studies that have utilized IVIs of anti-VEGF have shown comparable and potentially superior outcomes to PRP [8, 9]. However, both PRP and IVIs of anti-VEGF require patient adherence to follow-up visits in order to evaluate the response to therapy and the need for further interventions that prevent disease progression and vision loss. Causes of loss to follow-up (LTFU) from the patients' perspective cannot be elucidated unless some of the patients in this cohort eventually resume care. To our knowledge, this is the first study to address the causes of PDR patients LTFU after IVIs and/or PRP using questionnaire analysis.

This study aims to assess the clinical presentation of PDR patients LTFU when they eventually resume follow-up and assesses the causes of LTFU from the patients' perspective.

## 2. Materials and Methods

This study was reviewed and approved by the Medical Research and Ethics Committee of the Faculty of Medicine at Assiut University (Assiut, Egypt). Written informed consent was obtained from all patients after the nature/purpose of the study and risks/benefits of study participation were explained. All study conduct adhered to the tenets of the declaration of Helsinki.

### 2.1. Study Population

This prospective cohort study was conducted between May 25, 2013, and June 5, 2018, and included treatment-naïve patients who had developed PDR in one eye with a best corrected visual acuity (BCVA) ranging from 20/22 to 20/69, as determined by the Snellen equivalent. Patients were allocated to receive PRP, IVIs of anti-VEGF, or a combination of both procedures. Treatment decisions for each patient were guided by careful consideration of relative advantages of each treatment and the anticipated compliance with follow-up and treatment recommendations. A single retina specialist (M.S.) performed all laser and injection procedures at the Retina Outpatient Clinic in Assiut University Hospital (Assiut, Egypt). No new patients were recruited in the last 6 months of the observation period. Exclusion criteria were outlined as follows: (1) patients receiving follow-up ophthalmic care for their PDR with or without interventions at any other medical care provider during the observation period, as declared by the patients at any follow-up visit; (2) patients LTFU who did not resume follow-up until the end of the observation period; (3) patients needing PPV at first presentation or having additional retinal pathology; and (4) patients receiving their treatment procedure during December 2017 or having vitreous hemorrhage that failed to clear up by June 2018 but still ineligible candidates for PPV. LTFU was defined as missing any follow-up visit for any interval exceeding 6 months provided that patients eventually resumed care before the end of the study period (time zero was defined as the date of the missed follow-up visit).

### 2.2. Patient Characteristics and Clinical Assessment

Patient characteristics, including age and sex, were collected. Each patient received detailed complete ophthalmic examinations, including BCVA measurements, which were converted to a logarithm of the minimum angle resolution (LogMAR), intraocular pressure (IOP), slit lamp biomicroscopy, and indirect ophthalmoscopy, at the initial visit and at each follow-up visit. Fundus photography and fluorescein angiography were also performed at enrollment and when indicated during the follow-up period. The number of PRP sessions and IVIs of anti-VEGF and the need for PPV were also recorded. For LTFU subjects, a convenient treatment plan was established when care had resumed.

### 2.3. Subject Questionnaire

Subjects in the LTFU group were asked to complete an 8-item questionnaire regarding the reason(s) for missing their follow-up appointment. The questionnaire items were carefully chosen based on pilot discussions with subjects that had been in similar situations before conducting the study. Subjects were reminded that their answers would remain confidential and would not influence their future medical care. For patients with reading difficulties, the questionnaire was vocally administered. The questionnaire asked about the following potential causes for LTFU: (1) lack of information provided by medical care providers on follow-up need and/or date, (2) lack of concern and/or compliance (self-reported by subject), (3) lack of trust in and/or satisfaction with treatment, (4) lack of treatment affordability, (5) difficulty with transportation, (6) other disabling conditions (comorbidity) that hindered appointment attendance, (7) lack of a social support system, and (8) employment obligations. Because discussing treatment affordability and the potential lack of a support system may have upset some subjects, these questions were placed at the end of the survey to establish subject trust and to prevent emotional distress from confounding responses to the other questions. Subjects rated the impact of each item using a 5-point scale: 1 = “not significant at all” and 5 = “strongly significant cause.” Only completed surveys were used for analyses.

### 2.4. Statistical Analysis

Statistical tests were performed using SPSS, version 24 (SPSS, Inc., Chicago, IL, USA). Continuous variables are presented as mean ± standard deviation, and the frequency distributions of categorical variables were recorded. Age, sex, and type of intervention were used as categorical risk factors, and the differences in the rates of these factors for LTFU were assessed using chi-square tests. Univariate logistic regression was used to determine the odds of LTFU based on age, sex, and the type of intervention used. Factors with a *P* value < 0.1 were then used in a multivariate logistic regression model to determine the adjusted odds ratios for each risk factor. A *t*-test was used to compare the mean LogMAR BCVA between compliant patients group and LTFU group. Need for PPV was assessed in patients who were followed up and those who were LTFU; this information was analyzed in relation to risk factors (age, sex, and interventions) using a chi-square test. Spearman's rank correlation coefficients were used to assess correlations between the answer scale given for each question and the scales given for other questions as well as age and intervention used. Statistical significance was set at *P* < 0.05. Questionnaire responses were analyzed by mode of answers and frequencies.

## 3. Results

**Figure 1:**
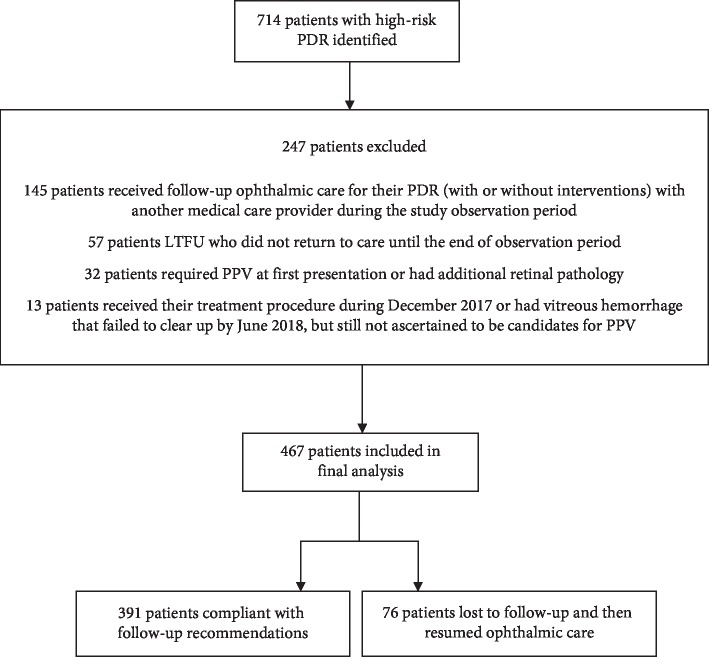
Flowchart of patients with high-risk proliferative diabetic retinopathy (PDR) included in the final analysis. PDR = proliferative diabetic retinopathy, LTFU = loss to follow-up, PPV = pars plana vitrectomy.

A total of 714 high-risk PDR patients were initially identified, and 467 patients were eligible for final analysis (Figure 1). The mean subject age was 56.40 ± 9.7 years, and there was a total of 219 (46.9%) female subjects. At the time of enrollment, LogMAR BCVA was 0.23 ± 0.08 (Snellen equivalent, 20/34) (*n* = 467). One hundred eighty (38.5%) participants received only PRP, 90 (19.3%) received only IVIs of anti-VEGF, and 197 (42.2%) received both PRP and IVIs of anti-VEGF. Patients receiving PRP attended 3.0 ± 1.2 sessions, patients receiving only IVIs of anti-VEGF received 3.2 ± 1.6 injections, and patients receiving both PRP and IVIs of anti-VEGF attended 2.8 ± 1.3 sessions and received 2.0 ± 1.1 injections. The median (interquartile range (IQR)) follow-up time of patients receiving PRP, IVIs of anti-VEGF, and both PRP and IVIs with anti-VEGF was 1.5 years (IQR 0.8–3.2), 1.3 years (IQR 0.4–2.8), and 1.6 years (IQR 0.8–3.4), respectively. A total of 76 patients (16.3%) were LTFU, and 391 patients (83.7%) were followed up as recommended or had delays less than 6 months beyond their predetermined follow-up visit date. A total of 57 patients were also LTFU but did not resume care until the end of the observation period; therefore, they were excluded because we could not assess the impact of LTFU or conduct questionnaires that were needed to fulfill study requirements. No patients had multiple LTFU intervals. Baseline characteristics of all patients with PDR are summarized in Table 1.

### 3.1. Effects of Age, Sex, and Intervention Used on Loss-to-Follow-Up Rates

As age increased, rates of LTFU decreased: 23.9% of patients aged ≤50 years were LTFU, 17% of patients aged 51–60 years were LTFU, and 9.3% of patients aged ≥61 years were LTFU (*P*=0.005). There were no significant differences between rates of LTFU and sex (16.1% in men and 16.4% in women). There was a significant difference between LTFU and the type of intervention used (*P*=0.003). Specifically, 23 (12.8%) PRP patients were LTFU as compared to 8 (8.9%) patients that received IVIs of anti-VEGF and 45 (22.8%) patients that received both PRP and IVIs of anti-VEGF (*P*=0.03). The mean LTFU duration was 4.9 ± 3.7 months after the initial 6-month delay.  Table 2 shows the interventions used for treating patients LTFU after resuming care until the end of the observation period.

### 3.2. Effect of Loss to Follow-Up on Visual Acuity

There was a statistically significant difference (*P*=0.001) in baseline mean LogMAR BCVA between patients who were followed up, 0.22 ± 0.07 (20/33) (*n* = 391), and patients LTFU, 0.26 ± 0.10 (20/36) (*n* = 76). At the final follow-up appointment, differences in the mean LogMAR BVCA had statistically significant difference (*P*=0.001) between patients who were followed up, 0.31 ± 0.28 (20/40) (*n* = 391), and patients LTFU, 0.47 ± 0.42 (20/59) (*n* = 76).

Patients LTFU who were treated with PRP had a baseline BCVA of 0.26 ± 0.10 (20/36) and a final BCVA of 0.48 ± 0.39 (20/60) (*P*=0.01). Patients LTFU who were treated with IVIs of anti-VEGF had a baseline BCVA of 0.24 ± 0.07 (20/34) and a final BCVA of 0.20 ± 0.06 (20/31) (*P*=0.31). Patients LTFU who were treated with both PRP and IVIs of anti-VEGF had a baseline BCVA of 0.2 5 ± 0.08 (20/35) and a final BCVA of 0.51 ± 0.56 (20/59) (*P* < 0.001).

### 3.3. Subjects Requiring Pars Plana Vitrectomy

Forty-six out of the 467 total patients needed PPV (9.9%), and 32 of the 391 (8.2%) followed up subjects and 14 of the 76 LTFU subjects (18.4%) required PPV; this difference was statistically significant (*P*=0.01). Additionally, patients LTFU had 122% (*P*=0.03) greater odds to need PPV than patients followed up. Duration of LTFU was a highly significant risk factor in determining the need for PPV. Patients LTFU who did not need PPV had a mean delay of 10.6 ± 3.4 months after their follow-up dates; meanwhile, patients LTFU who needed PPV had a mean delay of 14.7 ± 3.5 months after their follow-up dates (*P*=0.001).

### 3.4. Multivariate Regression

Both age and type of intervention significantly influenced LTFU rates, as determined using a univariate model; therefore, this data was also analyzed using a multivariate model (Table 3). Specifically, the increased odds of LTFU for patients aged ≤50 years and patients aged 51 to 60 years were 253% (*P*=0.001) and 142% (*P*=0.01), respectively, when compared with patients aged ≥61 years. Patients receiving both PRP and IVIs of anti-VEGF had 243% (*P*=0.003) greater odds of LTFU when compared with patients receiving IVIs of anti-VEGF.

### 3.5. Patient Questionnaire

A total of 76 LTFU subjects were asked to complete the LTFU cause questionnaire, but 3 subjects refused and 1 subject did not answer all of the questions. Therefore, 72 subjects were included in the subjective LTFU cause analyses. The mode of answers to all 8 questions was “not significant at all.” The LTFU cause was reported as “lack of concern/compliance” in 23 subjects (32%), “lack of treatment affordability” in 21 subjects (29.2%), “other disabling comorbidities” in 21 subjects (29.2%), “employment obligations” in 21 subjects (29.2%), “lack of trust/satisfaction with treatment” in 20 subjects (27.8%), “transportation difficulties” in 10 subjects (13.9%), and “lack of social support” in 9 subjects (12.5%). No subject answered that “lack of information” was a cause of LTFU.

Spearman's correlation analyses revealed significant and positive correlations between BCVA loss and subject lack of trust/satisfaction with treatment (rs = 0.46, *P* < 0.001), unaffordability and number of IVIs of VEGF (rs = 0.55, *P* < 0.001), transportation difficulties and lack of patient concern/compliance (rs = 0.34, *P*=0.003), disabling comorbidities and lack of social support (rs = 0.34, *P*=0.003), and lack of social support and age (rs = 0.39, *P* < 0.001). It also revealed significant and negative correlation between treatment unaffordability and employment obligations (rs = −0.44, *P* < 0.001), Disabling comorbidities and employment obligations (rs = 0.34, *P*=0.002), also between employment obligations, and age (rs = −0.03, *P*=0.002).

## 4. Discussion

The current study found an overall LTFU rate of 16.3% over approximately 5 years in patients who received PRP and/or IVIs of anti-VEGF to treat PDR. However, this figure represents patients who resumed care during the study period. When we include the patients who were excluded from analysis, the actual total rate of the LTFU patient cohort during the observation period rises to 25.4%. This relatively high rate occurred even though this study had a dedicated study coordinator and facility and optimized visit timing for each patient. In a randomized clinical trial, Gross et al. [8] found an average LTFU rate of 16.7%, 19%, and 20% at 2, 4, and 5 years, respectively (excluding deaths and withdrawals). Sivaprasad et al. [9] found an LTFU rate of 4% at 1 year (excluding deaths, randomization in errors, and exclusion due to eligibility after enrollment). In contrast, Subash et al. [10] found an LTFU rate of 12% at 6 months. Furthermore, Obeid et al. [11] reported an LTFU rate that exceeded 20% at 4 years in a retrospective cohort study. They suggested that a selection bias may explain the high LTFU rate because more concerned and compliant patients chose to participate in prospective trials. Approximately 60% of PDR patients respond to PRP (retinal neovascularization regression) within 3 months of treatment completion [4]. However, new retinal vessels may continue to grow after the first PRP session in one-third of patients [12]. Therefore, vitreous hemorrhage may lead to vision loss and prevent further PRP sessions in these patients. In contrast, IVIs of anti-VEGF can result in rapid retinal neovascularization regression after a single injection [13]. However, IVI efficacy has a relatively short duration and new vessels recur in 93% of eyes after 12 weeks [14, 15]. The effects of PRP last much longer [15], but many patients eventually require supplemental therapy [8, 9, 16, 17]. In our study, patients with persistent active PDR routinely underwent top-up PRP and IVI by the same treating retinal specialist who was masked to the study objectives. We observed a higher LTFU rate in subjects who underwent PRP than in those who underwent IVIs of anti-VEGF. Furthermore, this rate was even higher in patients who underwent both PRP and IVIs of anti-VEGF. Considering that IVIs are less painful than PRP [18], patients facing multiple PRP sessions are more likely to miss follow-up appointments than patients facing multiple IVIs. In addition, patients receiving IVIs are generally more satisfied with treatment than patients receiving PRP or a combination of interventions [8, 9, 18]. In addition to being painful, PRP is also associated with an increased risk of macular edema, peripheral visual field loss, and vitreous hemorrhage [8–10, 19], which leads to lower patient satisfaction as shown in our results. Our results also showed a positive correlation between the number of IVIs and treatment unaffordability; this may explain the higher LTFU rate in subjects who underwent both PRP and IVI.

There was a trend towards a decrease in rate of LTFU as age increased; the highest LTFU rate was observed among subjects who were younger than 51 years of age. Because our LTFU cause questionnaire also revealed a negative correlation between age and employment obligations, we believe that older patients are more likely to follow up than younger patients because of time constraints. However, we also identified a negative correlation between employment obligation and presence of comorbidities; therefore, sick leave benefits may help alleviate this issue across all age groups. Older patients tend to feel less pain during PRP than younger patients [20]; this was consistent with our results that showed lower LTFU rates in older subjects undergoing PRP than in younger subjects undergoing PRP. Our study showed that LTFU had no statistically significant influence on BCVA in subjects receiving IVI with anti-VEGF compared with subjects receiving PRP only and subjects receiving PRP and IVI with anti-VEGF. This can be explained by LTFU mean duration in this group which was significantly less (<0.001) compared to the other 2 groups highlighting the burden of prolonged LTFU duration on the final visual acuity of those patients.

The current study showed that the main LTFU cause (32% of LTFU cases) was “lack of concern/compliance.” Therefore, better education that highlights the importance of follow-up may slow or reverse the increasing LTFU rates [21]. More specifically, the importance of timely interventions, even when vision is good, should be stressed to PDR patients. Interestingly, none of our subjects reported “lack of information” as a cause of LTFU. This may have prevented a possible further increase in LTFU rates due to lack of information. Therefore, effective communication should be established between a patient's ophthalmologist and general physician [22] to further reduce LTFU rates. Lastly, low-cost mobile phone text message reminders should be used because these have been shown to significantly improve patient compliance and satisfaction [23].

Although anti-VEGF agents have been included in the World Health Organization list of essential medicines, the access, availability, and administration of anti-VEGF agents are erratic and may be financially unsustainable in developing countries with low- or intermediate-resource settings [24]. Importantly, most available evidence-based guidelines for diabetic retinopathy (DR) management are based on country-specific requirements. For instance, some countries may focus on one aspect of DR care, such as diabetic macular edema [25]. This restricts PDR treatments to PRP for many patients on national health insurance plans, decreases treatment satisfaction, and exposes patients to PRP-associated risks and discomfort [8, 9]. Unsatisfied patients often do not trust their medical care providers, which can lead to higher LTFU rates. Therefore, thorough, simple, and complete patient education beyond the procedural consent form should be provided to broaden patient awareness of the condition and how it is managed. Policies and services that increase health care quality and accessibility (e.g., providing transportation to medical appointments and improving medical staff's communication skills) can also improve patient trust [26]. The positive correlation between difficult transportation and lack of patient concern/compliance observed in the current study also suggests that providing transportation to medical appointments may increase patient compliance and decrease the need for social support. Egyptian diabetic patients receive their health care through various channels, including free government programs, university programs, medical care organizations, the national health insurance system, and private providers [27]. These programs can help mitigate out-of-pocket expense to between 0% and 30% of treatment costs. This is particularly important for PDR patients who require multiple IVIs. However, national health insurance systems frame government laws and regulations around health care and due to financial dependence on the government, treatment protocols may be influenced by a country's financial policies. Therefore, the high cost of PDR treatment remains a concern [28]. A recent cost analysis revealed that although the cost differential between intravitreal ranibizumab and PRP may not be significant at the 2-year follow-up, over a lifetime this differential may increase significantly [29]. Other less expensive anti-VEGF agents may one day decrease this cost differential [30]. The current study showed that high lifetime treatment cost is also a concern to patients, as evidenced by the significant positive correlation between treatment unaffordability and the number of IVIs. Furthermore, the significant negative correlation between treatment unaffordability and the presence of employment obligations revealed that unemployed patients with little or no access to health insurance are particularly vulnerable to becoming LTFU because of high treatment costs.

Srikanth [31] reported that patients remain in the PDR state for the longest time period (nearly 8 years) before transitioning into blindness. However, Negretti et al. [32] stressed that the national guidelines for scheduled follow-up remain important due to the occasional patient who is at a very high risk of vision loss and the implications that the proper timely assessment has on patients' medical and ocular condition. It was also stressed that this group of patients is more often socially deprived, with an increased likelihood of other medical conditions, and they often have a very poor attendance history [33]. Our findings revealed significant positive correlations between lack of social support and presence of other disabling comorbidities and lack of social support and age, further stressing the vulnerability of these patients to LTFU.

Our study had several limitations. First, there is no ideal time interval for defining LTFU because PRP and IVI follow-up schedules can vary widely between PDR patients. Second, treatment decisions were not randomized in this study, which may have introduced a selection bias. Third, the study results cannot be generalized internationally. Lastly, the anti-VEGF agent used for IVI therapy varied between subjects and this was not considered in our analyses. Despite these limitations, this prospective study shows the relatively large proportion of PDR patients who were LTFU after treatment over approximately 5 years of follow-up and elucidates drawbacks as well as possible causes of LTFU in a proportion of these patients. These findings may be useful for determining the management of this category of patients at risk of being LTFU. If noncompliant subjects and dropouts are excluded from the final analysis, important prognostic differences may be created among treatment groups. Moreover, subjects may be noncompliant or may drop out from the study due to their response to treatment [34].

In conclusion, PDR patients who become LTFU are at a considerable risk of vision loss. Therefore, it is imperative to address potential LTFU causes in each patient and customize interventions to mitigate this risk. Further studies in other countries and in different socioeconomic populations are needed to confirm our findings and understand LTFU risk factors in socially, economically, and racially diverse populations.

## Figures and Tables

**Table 1:** Characteristics of all patients with proliferative diabetic retinopathy enrolled in the study according to follow-up status.

	Followed up	LTFU	All subjects	*P* value
Number of eyes (%)	391 (83.7%)	76 (16.3%)	467	—
Baseline LogMAR BCVA (Snellen equivalent)	0.22 ± 0.07 (20/33)	0.26 ± 0.10 (20/36)	0.23 ± .08 (20/34)	0.001
Age (yrs)	57.11 ± 9.83	52.78 ± 8.25	56.40 ± 9.72	0.001
Gender, *n* (%)				0.93
Male	208 (83.9%)	40 (16.1%)	248	
Female	183 (83.6%)	36 (16.4%)	219	
Age category (yrs), *n* (%)				0.005
≤50 years	89 (76.1%)	28 (23.9%)	117	
51–60 years	166 (83.0%)	34 (17.0%)	200	
≥61 years	136 (90.7%)	14 (9.3%)	150	
Procedure, *n* (%)				0.003
IVI	82 (91.1%)	8 (8.9%)	90	
PRP	157 (87.2%)	23 (12.8%)	180	
IVI + PRP	152 (77.2%)	45 (22.8%)	197	
LogMAR BCVA at final follow-up (Snellen equivalent)	0.31 ± 0.28 (20/40)	0.47 ± 0.42 (20/59)	0.43 ± 0.32(20/43)	0.001

Data presented mean ± standard deviation and *n* (%) as applicable. For LTFU group, interventions represented were done before point of LTFU. BCVA = best corrected visual acuity, IVI = intravitreal injection, LogMAR = logarithm of the minimum angle resolution, LTFU = loss to follow-up, and PRP = panretinal photocoagulation.

**Table 2:** Interventions used for treating proliferative diabetic retinopathy in patients lost to follow-up after resuming care until the end of the observation period.

PDR treatment	Number of patients LTFU	Duration of resumed follow-up to final visit (months)	Duration of resumed follow-up to final visit (months)	Duration of resumed follow-up to final visit (months)	Duration of resumed follow-up to final visit (months)
		PRP	IVI (s)	PPV	
IVI	8	4	5	2	7.3 ± 2.5
PRP	23	12	9	3	4.1 ± 1.7
PRP + IVI (s)	45	19	32	9	7.8 ± 1.1

IVI = intravitreal injection, LTFU = loss to follow-up, PRP = panretinal photocoagulation, PPV = pars plana vitrectomy, and PDR = proliferative diabetic retinopathy.

**Table 3:** Univariate and multivariate logistic regression model evaluating age and procedure used as potential risk factors associated with loss to follow-up in patients with proliferative diabetic retinopathy.

	LTFU *n* (%)	Univariate model		Multivariate model	
		Odds ratio (95% CI)	*P* value	Odds ratio (95% CI)	*P* value
Procedure					
IVI	8 (8.9%)	Reference		Reference	
PRP	23 (12.78%)	1.50 (0.64–3.51)	0.34	1.47 (0.62–3.47)	0.37
PRP + IVI	45 (22.8%)	3.04 (1.36–6.74)	0.006	3.43 (1.52–7.73)	0.003

Age, years					
≥61	14 (9.3%)	Reference		Reference	
51–60	34 (17.0%)	1.99 (1.02–3.85)	0.04	2.42 (1.23–4.78)	0.01
≤50	28 (23.9%)	3.05 (1.52–6.12)	0.002	3.53 (1.73–7.19)	0.001

LTFU = loss to follow-up, CI = confidence interval, IVI = intravitreal injection, and PRP = panretinal photocoagulation.

## Data Availability

The data used to support the findings of this study are included within the article.

## Abbreviations

LTFU: Loss to follow-up
IVIs: Intravitreal injections
BCVA: Best corrected visual activity
DR: Diabetic retinopathy
IQR: Interquartile range
IOP: Intraocular pressure
Log MAR: Logarithm of the minimum angle resolution
PRP: Panretinal photocoagulation
PPV: Pars planavitrectomy
PDR: Proliferative diabetic retinopathy
VEGF: Vascular endothelial growth factor.
